# Pan-cancer analysis identifies DDX56 as a prognostic biomarker associated with immune infiltration and drug sensitivity

**DOI:** 10.3389/fgene.2022.1004467

**Published:** 2022-12-07

**Authors:** Zhaohui Ruan, Yuetong Zhang, Qi Quan, Jiaxin Jiang, Qianyu Wang, Yujing Zhang, Roujun Peng

**Affiliations:** ^1^ VIP Department, State Key Laboratory of Oncology in South China, Guangdong Key Laboratory of Nasopharyngeal Carcinoma Diagnosis and Therapy, Sun Yat-sen University Cancer Center, Guangzhou, China; ^2^ Radiation Oncology, State Key Laboratory of Oncology in South China, Guangdong Key Laboratory of Nasopharyngeal Carcinoma Diagnosis and Therapy, Sun Yat-sen University Cancer Center, Guangzhou, China

**Keywords:** DDX56, pan-cancer, multi-omics data, bio-marker, survival

## Abstract

DDX56, a member of the RNA helicase family, is upregulated in colon adenocarcinoma, lung squamous cell carcinoma, and osteosarcoma. However, the relationships between DDX56 and other tumors are not clear, and the molecular mechanism of its action is not fully understood. Here, we explore the biological functions of DDX56 in 31 solid tumors and clarify that DDX56 can promote oncogenesis and progression in multiple tumor types based on multi-omics data. Bioinformatics analysis revealed that the cancer-promoting effects of DDX56 were achieved by facilitating tumor cell proliferation, inhibiting apoptosis, inducing drug resistance, and influencing immune cell infiltration. Furthermore, we found that copy number alterations and low DNA methylation of *DDX56* were likely to be related to aberrantly high *DDX56* expression. Our results suggest that DDX56 is a potential pan-cancer biomarker that could be used to predict survival and response to therapy, as well as a potential novel therapeutic target. We validated some of our results and illustrated their reliability using CRISPR Screens data. In conclusion, our results clarify the role of DDX56 in the occurrence and development of multiple cancers and provide insight into the molecular mechanisms involved in the process of pathogenesis, indicating a direction for future research on DDX56 in cancers.

## Introduction

Cancer poses a great threat to human health and is a leading cause of death, with more than 19.3 million people diagnosed with cancer and more than 10.0 million deaths each year (Sung et al., 2021). The identification of key molecular targets in various cancers has helped to enhance treatment effects and improve the prognosis of cancer patients. For instance, sorafenib, which can inhibit multiple tyrosine kinases including VEGFR1, VEGFR2, KIT, and PDGFR-α, is widely used in hepatocellular carcinoma (HCC) and renal cell cancer (Bedard et al., 2020). In breast cancer, individual treatment strategies targeting molecular subtypes have dramatically improved survival outcomes in 70–80% of patients (Loibl et al., 2021). These reports show that identification of critical molecules can lead to innovation in cancer therapeutic strategies, with massive application potential. Therefore, the exploration of new key molecules and underlying mechanisms is of great significance.

In addition to molecular targeted therapies, immunotherapy is a prospective treatment for multiple cancer types. The focus of immunotherapy has shifted from the tumor itself to the host’s immune system and tumor microenvironment, with the aim of mobilizing immune cells to discern and eventually eliminate cancer cells (Sharma et al., 2017). Immunotherapy based on immune checkpoint inhibitors has dramatically changed management strategies for various advanced cancers, including non-small-cell lung cancer, extensive small-cell lung cancer, HCC, and classical Hodgkin lymphoma) (Lee et al., 2022). Combination therapy with anti-CTLA4 and anti-PD-1 checkpoint inhibition is an effective option for advanced melanoma and unresectable malignant pleural mesothelioma (Baas et al., 2021; Carlino et al., 2021). Treatment targeting LAG3 has also shown good response (Andrews et al., 2017). However, not all patients can benefit from immunotherapy, and there is a lack of effective markers to predict response to immunotherapy. Therefore, it is urgent to screen more therapeutic targets and identify predictive biomarkers of immunotherapy.

DDX56 is a member of the DEAD box RNA helicase family that plays a key part in various RNA-related biological processes (Cordin et al., 2006; Linder and Jankowsky, 2011). Previous studies have shown that DDX56 can promote the occurrence and development of colon cancer, osteosarcoma, glioblastoma, and lung squamous cell carcinoma (Kouyama et al., 2019; Zhu et al., 2020; Pryszlak et al., 2021; Wu et al., 2021). However, the role of DDX56 in other tumors has not been reported. In addition, it has been reported that other members of DEAD box RNA helicase family can induce resistance of tumor cells to chemotherapeutic agents (Park et al., 2018; Mani et al., 2020). Whether DDX56 contributes to tumor progression or could be used as a biomarker remains to be determined. Here, based on bioinformatics analysis of multi-omics data, we illustrate that DDX56 is involved in the occurrence and development of multiple tumors. Further, we conduct co-expression and enrichment analyses of the biological functions of DDX56 in various solid cancers. We also investigate the potential associations between *DDX56* expression and immune infiltration levels and immune-related markers. Finally, we explore the possible mechanisms of high DDX56 expression in tumor tissues. Our results demonstrate the role of DDX56 in oncogenesis in multiple tumors and its potential to serve as a therapeutic target and prognostic indicator.

## Materials and methods

### Data collection and expression analysis

We compared *DDX56* RNA expression among different tissues using RNA sequencing (RNA-seq) datasets from The Cancer Genome Atlas (TCGA). RNA-seq data (TPM) and related clinical data were downloaded from UCSC Xena (http://xena.ucsc.edu/) (Goldman et al., 2020). The data corresponded to 31 solid tumor types: adrenocortical carcinoma (ACC), bladder urothelial carcinoma (BLCA), breast invasive carcinoma (BRCA), cervical squamous cell carcinoma and endocervical adenocarcinoma (CESC), cholangiocarcinoma (CHOL), colon adenocarcinoma (COAD), esophageal carcinoma (ESCA), glioblastoma multiforme (GBM), head and neck squamous cell carcinoma (HNSC), kidney chromophobe (KICH), kidney renal clear cell carcinoma (KIRC), kidney renal papillary cell carcinoma (KIRP), brain lower grade glioma (LGG), liver hepatocellular carcinoma (LIHC), lung adenocarcinoma (LUAD), lung squamous cell carcinoma (LUSC), mesothelioma (MESO), ovarian serous cystadenocarcinoma (OV), pancreatic adenocarcinoma (PAAD), pheochromocytoma and paraganglioma (PCPG), prostate adenocarcinoma (PRAD), rectum adenocarcinoma (READ), sarcoma (SARC), skin cutaneous melanoma (SKCM), stomach adenocarcinoma (STAD), testicular germ cell tumors (TGCT), thyroid carcinoma (THCA), thymoma (THYM), uterine corpus endometrial carcinoma (UCEC), uterine carcinosarcoma (UCS), uveal melanoma (UVM). Using the “Gene” module of TISCH (http://tisch.comp-genomics.org/search-gene/), we determined the RNA expression of *DDX56* in multiple cell types based on single-cell RNA-seq data (Sun et al., 2021). UALCAN carried out a comparative analysis of protein expression (http://ualcan.path.uab.edu/index.html) (Chandrashekar et al., 2017). Only tumors with matched normal tissue data were used for differential analysis. We defined clinical stages as follows: early stages, TNM stages I/II; advanced stages, TNM stages III/IV.

### Survival analysis

Univariate Cox regression was used to assess the prognostic significance of DDX56 across cancer types. Multivariate Cox regression was used to identify independent prognostic factors. The surv_cutpoint function (from R package “survminer”, https://github.com/kassambara/survminer) was used to determine the optimal cutoff values of *DDX56* expression level. Only solid tumor data with complete clinical information were included in the survival analysis.

### Co-expression and functional enrichment

Genes co-expressed with DDX56 were screened by calculating the Spearman correlation coefficient between DDX56 and all other genes in each cancer. The Benjamini–Hochberg method was used to decrease the false discovery rate (adjusted *p*-value). To gain functional and mechanistic insights regarding DDX56, we performed a pre-ranked gene set enrichment analysis (GSEA) (Subramanian et al., 2005) based on Hallmarker’s gene sets, which were downloaded from the MSigDB database (https://www.gsea-msigdb.org/gsea/msigdb/) (Liberzon et al., 2015). The genes with significant correlation coefficients (adjusted *p* < 0.05) were sorted according to Spearman correlation coefficient and then used in GSEA.

### Validation of functional enrichment results of DDX56 based on CRISPR screens

The CRISPR pooled libraries consist of thousands of plasmids, each containing multiple gRNAs for each target gene (Shalem et al., 2014). In a CRISPR screening experiment, target cells are treated with the pooled library to create a population of mutant cells that are then screened for a phenotype of interest. Essential genes for specific phenotypic screens can be curated by comparing sgRNA abundance. Therefore, the database includes research information on whether a particular gene is essential for a certain phenotype in a particular tumor cell line (Joung et al., 2017). Compiled CRISPR screen data were obtained from the Biological General Repository for Interaction Datasets (BioGRID) (https://orcs.thebiogrid.org/) (Oughtred et al., 2019). Data from proliferation-based CRISPR screens in solid tumor cancer cell lines were selected. These datasets were used to verify the mitogenic activity of DDX56. Resistance-related CRISPR screening evidence was also retrieved from the BioGRID database. The methods were as previously described except that the studies chosen focused on the “response to chemicals” phenotype.

### Drug sensitivity analysis

Half-maximal inhibitory concentration (IC50) data and associated RNA-seq data from multiple cancer cell lines were obtained from the CellMiner database (https://discover.nci.nih.gov/cellminer/) (Reinhold et al., 2012). We conducted Spearman correlation analysis between *DDX56* expression and drug IC50 in order to investigate the relationship between *DDX56* expression and drug sensitivity.

### Profiling tumor-infiltrating immune cells (TILs)

Proportions of TILs were estimated using the CIBERSORT function (https://cibersort.stanford.edu/). (Newman et al., 2015; Chen et al., 2018). Based on the Spearman correlation coefficient, we assessed the statistical correlation between *DDX56* expression level and the proportion of TILs.

### Prediction analysis for immunotherapy

We evaluated the value of DDX56 in predicting immunotherapy response in an immune checkpoint blockade therapy cohort. The transcriptomic data and relevant clinical data from this cohort were obtained from dbGaP (phs000452) (Liu et al., 2019a). Univariate Cox regression was used to assess the prognostic significance of DDX56.

### Gene mutation and methylation analysis of DDX56 and identification of related transcription factors

We conducted gene mutation analysis on cBioportal (https://www.cbioportal.org/) based on TCGA Pan-Cancer Atlas data (Cerami et al., 2012). The correlation of *DDX56* RNA expression level with copy number variation (CNV) was evaluated by MEXPRESS (https://mexpress.be/) (Koch et al., 2019). The correlation between the expression data and the DNA methylation data was estimated by using MEXPRESS (https://mexpress.be/). Oncodb (http://oncodb.org/) was used to compare methylation profiles between tumor tissues and normal tissues (Koch et al., 2019; Tang et al., 2022). We used the Toolkit for CistromeDB (http://dbtoolkit.cistrome.org/) to predict which transcription factors had the greatest potential to enhance expression of DDX56 (Zheng et al., 2019).

### Statistical analysis

We compared non-normally distributed continuous variables using Wilcoxon rank-sum test and compared categorical variables using chi-square test between two groups. Kaplan–Meier and log-rank tests were conducted for survival analysis. Unless otherwise stated, all data analysis was performed in R (version 4.1.0). Unless otherwise specified, *p* < 0.05 was considered to indicate statistical significance in all analyses. The tumors involved in each analysis are recorded in Supplementary Table S1.

## Results

### Pan-cancer expression profiles of DDX56

**FIGURE 1:**
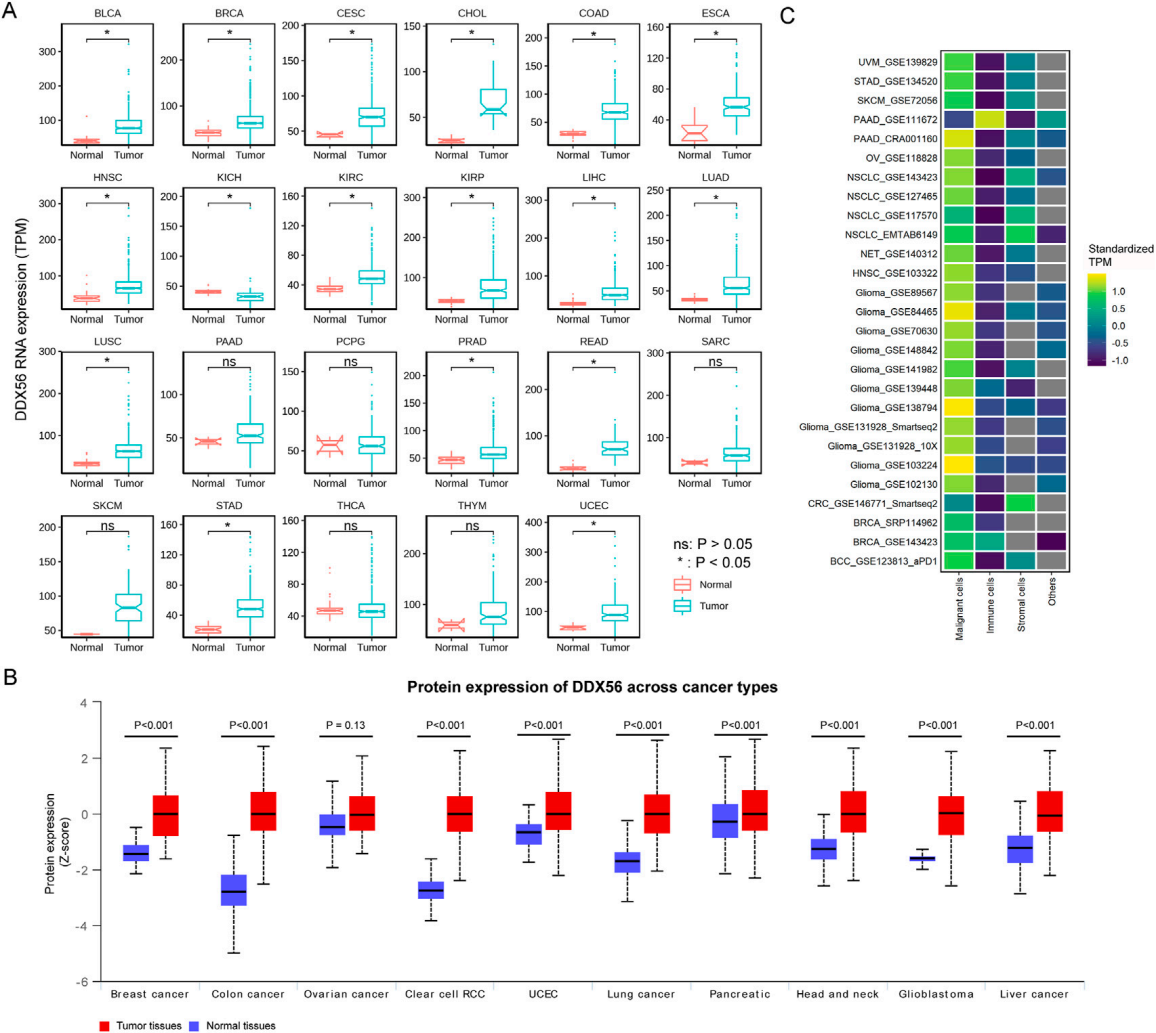
RNA and protein expression of DDX56 in various tumors. **(A)** Box plots showing RNA expression of *DDX56* in different tissues (Wilcoxon test). *P*-values are marked. **(B)** Box plots showing protein expression levels of DDX56 in different tissues (T test). *P*-values are marked. **(C)** Heat map showing RNA expression in different cell types of various tumors. TPM values were standardized for each tumor. The colors indicate the TPM value after standardization. The X-axis indicates single-cell RNA-seq datasets. The Y-axis indicates the cell type. Abbreviations: BLCA, bladder urothelial carcinoma; BRCA, breast invasive carcinoma; CESC, cervical squamous cell carcinoma and endocervical adenocarcinoma; CHOL, cholangiocarcinoma; COAD, colon adenocarcinoma; ESCA, esophageal carcinoma; GBM, glioblastoma multiforme; HNSC, head and neck squamous cell carcinoma; KICH, kidney chromophobe; KIRC, kidney renal clear cell carcinoma; KIRP, kidney renal papillary cell carcinoma; LIHC, liver hepatocellular carcinoma; LUAD, lung adenocarcinoma; LUSC, lung squamous cell carcinoma; PAAD, pancreatic adenocarcinoma; PCPG, pheochromocytoma and paraganglioma; PRAD, prostate adenocarcinoma; READ, rectum adenocarcinoma; SARC, sarcoma; SKCM, skin cutaneous melanoma; STAD, stomach adenocarcinoma; THCA, thyroid carcinoma; THYM, thymoma; UCEC, uterine corpus endometrial carcinoma; BCC, basal cell carcinoma; NET, Neuroendocrine tumor; CRC, colorectal cancer; RCC, renal cell carcinoma.

We compared *DDX56* RNA expression between different cancer tissues and matched normal tissues based on TCGA data. The pan-cancer analysis showed that the *DDX56* RNA expression level was higher in 16 solid cancers than in their matched normal tissues (Wilcoxon test, *p* < 0.05) (Figure 1A), consistent with findings in lung squamous cell carcinoma (LUSC), osteosarcoma (OV), and colon cancer (COAD) (Zhu et al., 2020; Pryszlak et al., 2021; Wu et al., 2021). UALCAN was used to determine the protein expression of DDX56 in different types of cancer. According to significant unique analyses (provided by UALCAN, Student’s t-test), DDX56 protein was significantly over-expressed in nine tumor types (*p* < 0.05, Figure 1B). An effective tumor biomarker and drug target requires specific and high expression in tumor cells compared with other components of the tumor microenvironment. Therefore, we analyzed pan-cancer single-cell RNA-seq data using the TISCH database and observed that *DDX56* was mainly expressed in tumor cells rather than immune cells, stromal cells, and others (Figure 1C), indicating the potential of DDX56 as a tumor biomarker and drug target.

### Prognostic value of DDX56 in multiple cancers

**FIGURE 2:**
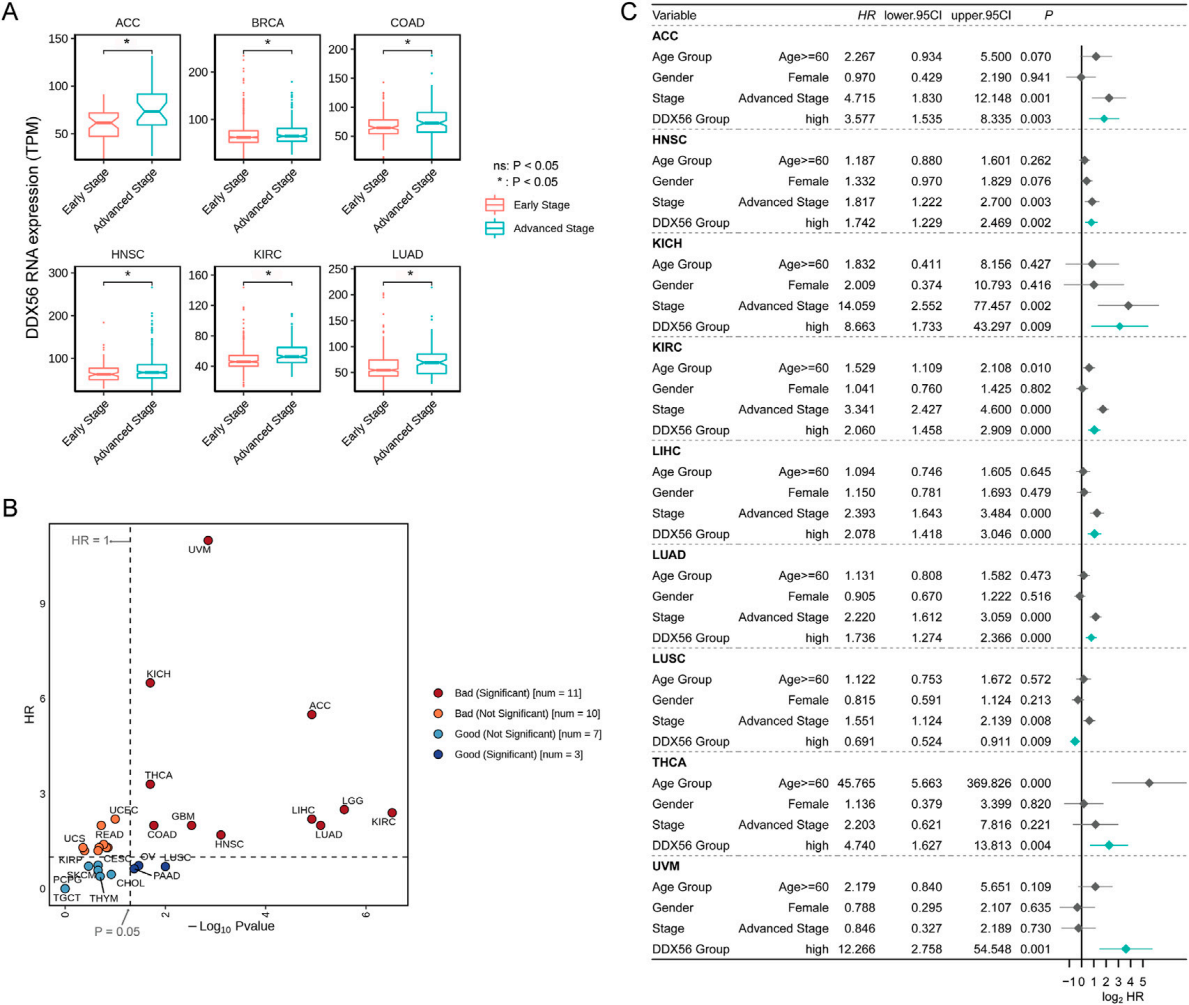
Prognostic value of DDX56 in various cancers. **(A)** Box plots showing RNA expression levels of *DDX56* at different clinical stages. For each tumor, stages Ⅰ and Ⅱ were classified as early stage; stages Ⅲ and Ⅳ were classified as advanced stage. **(B)** Scatter plot showing outcomes in cancer patients with different *DDX56* expression levels. Statistics were obtained by univariate Cox regression analysis. **(C)** Forest plot showing the results of multivariate Cox regression for multiple tumor types. Abbreviations: ACC, adrenocortical carcinoma; BRCA, breast invasive carcinoma; COAD, colon adenocarcinoma; GBM, glioblastoma multiforme; HNSC, head and neck squamous cell carcinoma; KICH, kidney chromophobe; KIRC, kidney renal clear cell carcinoma; LIHC, liver hepatocellular carcinoma; LUAD lung adenocarcinoma; LUSC, lung squamous cell carcinoma; MESO, mesothelioma; OV, ovarian serous cystadenocarcinoma; PAAD, pancreatic adenocarcinoma; PCPG, pheochromocytoma and paraganglioma; PRAD, prostate adenocarcinoma; READ, rectum adenocarcinoma; SARC, sarcoma; SKCM, skin cutaneous melanoma; STAD, stomach adenocarcinoma; TGCT, testicular germ cell tumors; THCA, thyroid carcinoma; THYM, thymoma; UCEC, uterine corpus endometrial carcinoma; UCS, uterine carcinosarcoma; UVM, uveal melanoma.

Next, we assessed the correlation between *DDX56* expression and the clinical features of patients. Although few significant differences were observed between groups in age or gender (Supplementary Figure S1), the expression level of *DDX56* was consistently higher in patients in the advanced stages than those in the early stages of diseases including ACC, BRCA, COAD, HNSC, KIRC, and LUAD (Wilcoxon test*, p* < 0.05, Figure 2A and Supplementary Figure S1). Further, we investigated the prognostic value of DDX56 at a pan-cancer level. The results showed that high *DDX56* expression was correlated with worse outcomes in 11 cancer types (univariate Cox regression, *p* < 0.05, hazard ratio >1) (Figure 2B and Supplementary Figure S2). Then, we implemented multivariate Cox regression analysis based on *DDX56* expression level and other clinical factors and found that DDX56 was an independent predictor for cancer prognosis in nine tumor types; moreover, worse clinical outcomes in patients with higher expression of *DDX56* were observed in eight tumor types, comprising ACC, HNSC, KICH, KIRC, LIHC, LUAD, THCA, and UVM (Figure 2C). These results suggest that *DDX56* high expression may be a significant predictor of prognosis and function as a promotor in multiple tumor types.

### DDX56 has a pro-proliferative property at the pan-cancer level

**FIGURE 3:**
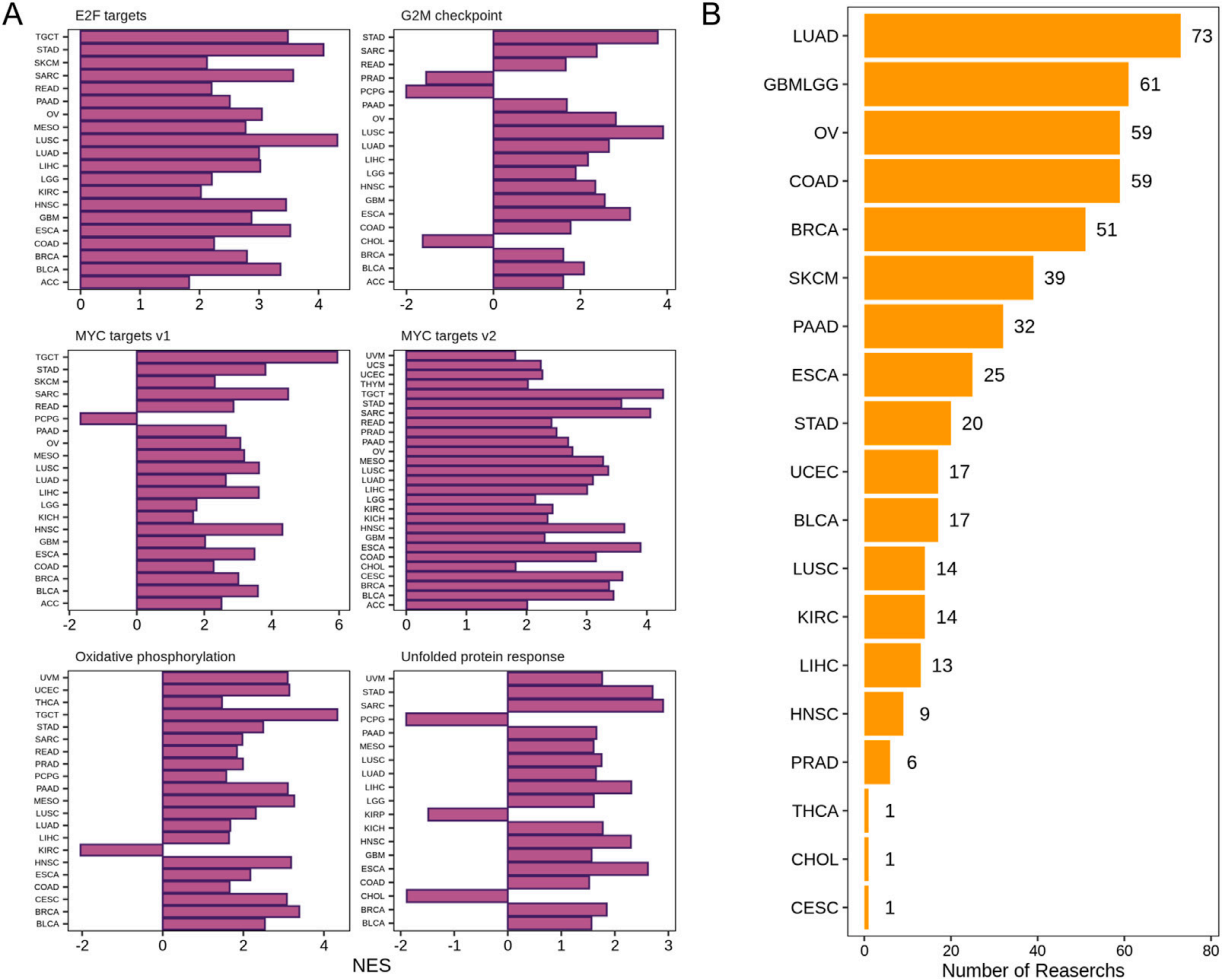
DDX56 positively modulates the proliferation of cancer cells. **(A)** GSEA enrichment results for DDX56 in multiple tumor types. Only pathways deemed to be significantly enriched based on GSEA (*p* < 0.05) are illustrated. **(B)** Multiple CRISPR screening studies verifying that DDX56 can positively regulate the proliferation of cancer cells. *X*-axis indicates the number of CRISPR screening studies supporting a pro-proliferative phenotypic role of this gene in tumor cell lines. *Y*-axis indicates the tumor type of the CRISPR screens studies. Abbreviations: ACC: adrenocortical carcinoma; BRCA: breast invasive carcinoma; COAD: colon adenocarcinoma; GBM: glioblastoma multiforme; HNSC: head and neck squamous cell carcinoma; KICH: kidney chromophobe; KIRC: kidney renal clear cell carcinoma; LIHC: liver hepatocellular carcinoma; LUAD: lung adenocarcinoma; LUSC: lung squamous cell carcinoma; MESO: mesothelioma; OV: ovarian serous cystadenocarcinoma; PAAD: pancreatic adenocarcinoma; PCPG: pheochromocytoma and paraganglioma; PRAD: prostate adenocarcinoma; READ: rectum adenocarcinoma; SARC: sarcoma; SKCM: skin cutaneous melanoma; STAD: stomach adenocarcinoma; TGCT: testicular germ cell tumors; THCA: thyroid carcinoma; THYM: thymoma; UCEC: uterine corpus endometrial carcinoma; UCS: uterine carcinosarcoma; UVM: uveal melanoma.

To explore the molecular mechanism of DDX56 in carcinogenesis, a network of genes co-expressed with DDX56 was first conducted in each tumor dataset, and then pathway enrichment analysis was performed using GSEA. The results revealed that DDX56 mainly participated in cell-proliferation-related signaling pathways, including G2/M checkpoint, MYC targets v1, MYC targets v2, and E2F targets (Figure 3A) (Liberzon et al., 2015). Concomitantly, we observed enrichment of tumor metabolism-related pathways including oxidative phosphorylation and unfolded protein response. In order to validate the possible pro-proliferation function of DDX56, we collected and analyzed CRISPR screens data from the BioGRID Open Respository of CRISPR Screens. CRISPR-based genetic screening is a powerful tool to identify the genes required for specific functions such as cell viability and chemical resistance. We found 512 CRISPR screens studies in 19 tumor types, which confirmed the effects of DDX56 on the proliferation of tumor cells at the pan-cancer level (Figure 3B and Supplementary Table S2).

### Correlations between DDX56 expression and multiple drug resistance

**FIGURE 4:**
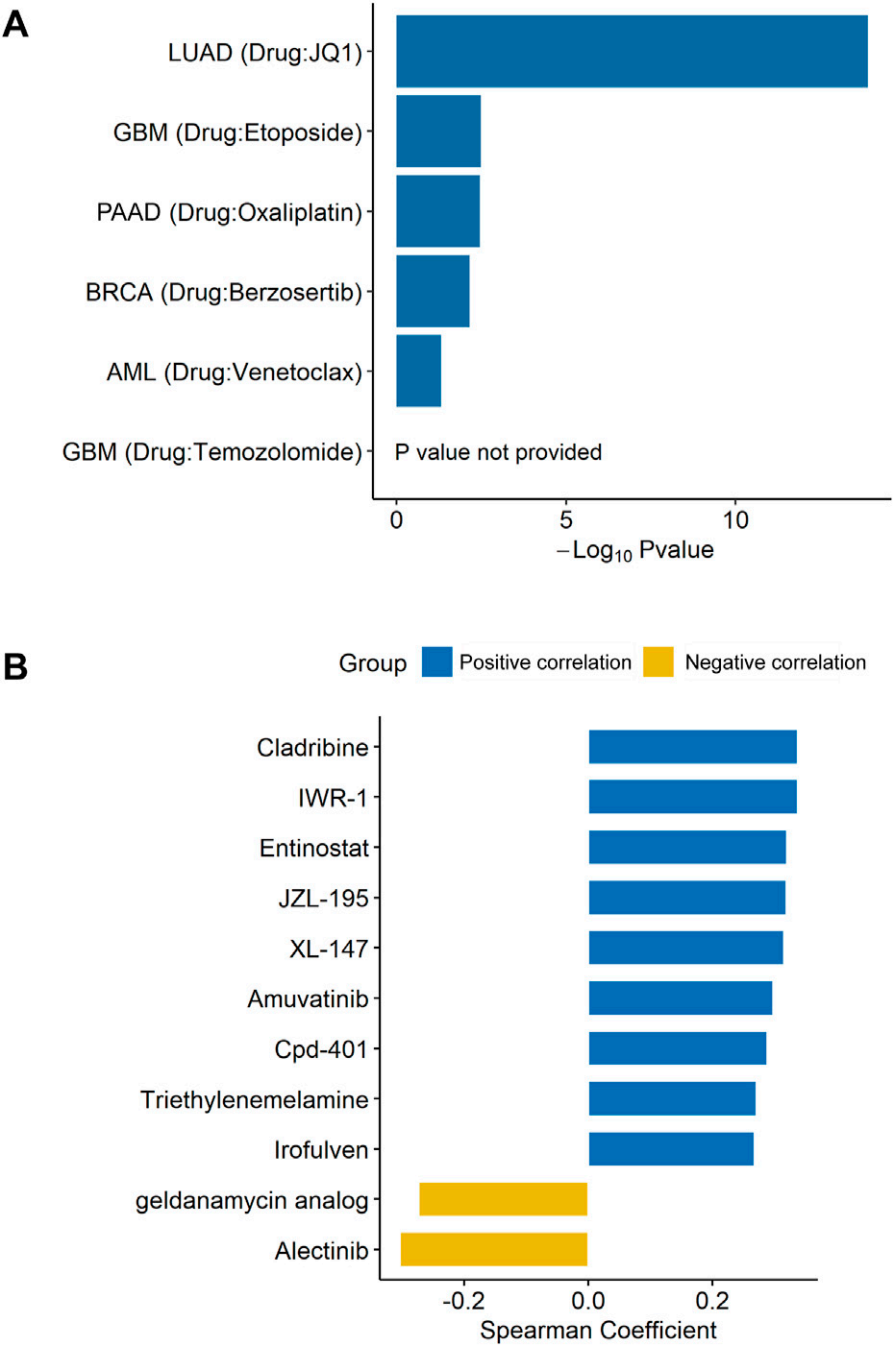
DDX56 promotes drug resistance in multiple tumor types. **(A)** Multiple CRISPR screening studies showing that DDX56 is associated with drug resistance. *Y*-axis indicates the tumor type and the associated drug. *X*-axis indicates the logarithm of the negative *p*-value of DDX56 in each CRISPR screening study. **(B)** Bar plot showing the Spearman coefficients for *DDX56* RNA expression and drug IC50. Eleven statistically significant results were obtained from 450 drugs in the screen. Abbreviations: LUAD: lung adenocarcinoma; GBM: glioblastoma multiforme; PAAD: pancreatic adenocarcinoma; BRCA: breast invasive carcinoma; AML: acute myeloid leukemia.

As the GSEA results had shown enrichment of unfolded protein response (Figure 3A), a chemoresistance-related pathway, (Bahar et al., 2019; Sisinni et al., 2019), we speculated that DDX56 was associated with chemotherapy resistance. To investigate whether DDX56 was relevant to drug resistance, we searched the CRISPR screens evidence in the BioGRID database. We found that DDX56 was an essential gene required for drug resistance in five different tumors (Figure 4A and Supplementary Table S3). Moreover, we obtained IC50 levels of all available drugs for tumor cell lines from the CellMiner database and calculated the Spearman correlation coefficient between the IC50 values and *DDX56* RNA expression in matched tumor cell lines. Our results revealed that high expression of DDX56 was correlated with increased resistance to cladribine, entinostat, amuvatinib, triethylenemelamine, irofulven, IWR-1, JZL-195, XL-147, and Cpd-401 (*p* < 0.05, Figure 4B). On the other hand, high expression of DDX56 could lead to enhanced sensitivity to a geldanamycin analog and alectinib (*p* < 0.05, Figure 4B).

### Correlations between DDX56 expression and immune infiltration levels in cancers

**FIGURE 5:**
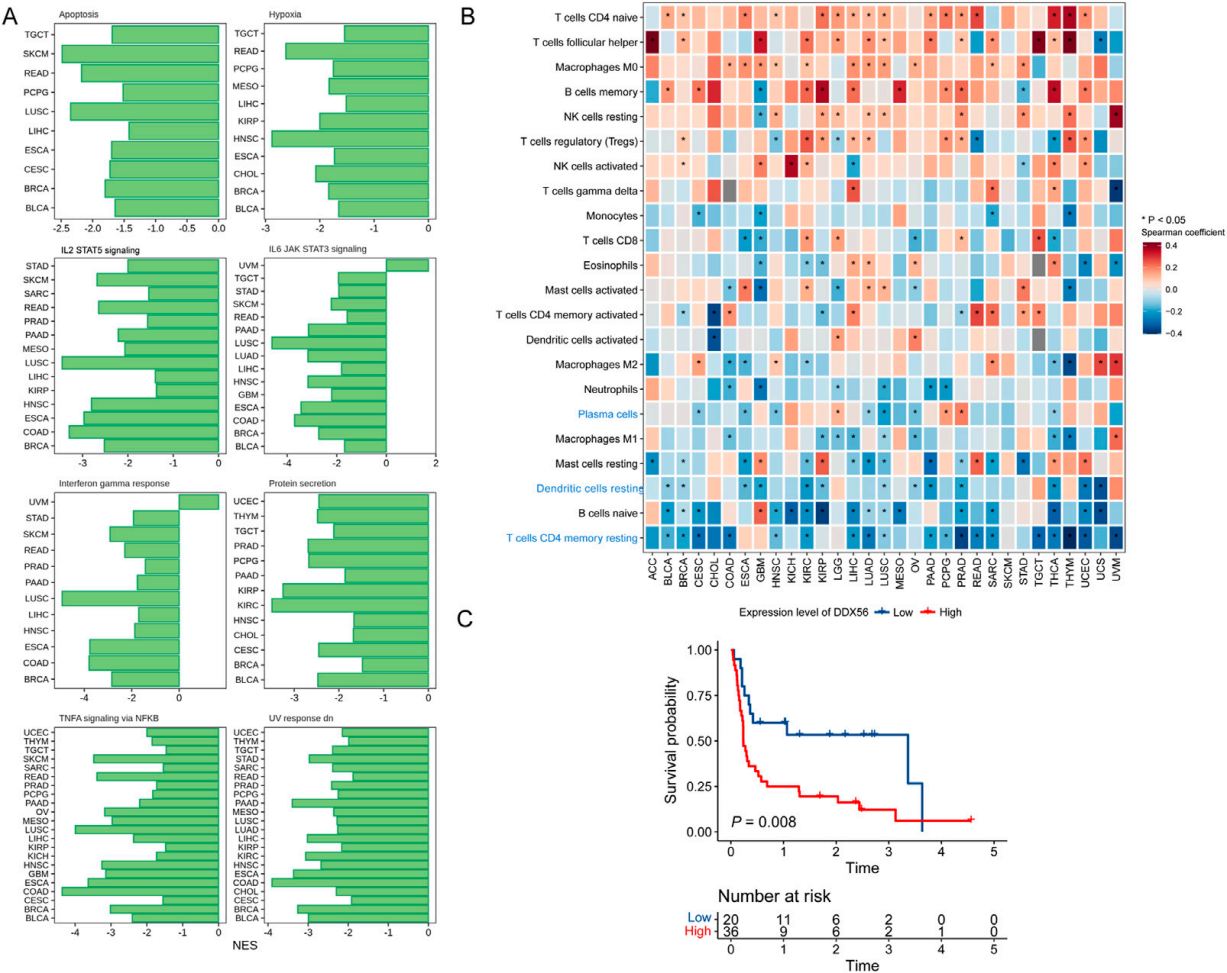
Relationships of DDX56 with tumor immune features. **(A)** GSEA enrichment results for DDX56 in multiple tumor types. Only pathways deemed significant enriched based on GSEA (*p* < 0.05) are illustrated. **(B)** Heat map showing Spearman coefficients for *DDX56* RNA expression and proportions of tumor-infiltrating immune cells. **(C)** Kaplan–Meier plots showing worse clinical outcome in immunotherapy cohort patients with higher expression of *DDX56*. Univariate Cox regression was used to assess the statistics.

The GSEA results suggested that DDX56 may have a critical role in inhibiting cell apoptosis and suppressing antitumor immunity (IL-2 STAT5 signaling, IL-6 JAK STAT3 signaling, and interferon gamma response, Figure 5A) (Liberzon et al., 2015). To further explore the potential relationships between DDX56 and immune cells, we examined the correlations between *DDX56* and several immune cell markers including immune cells (*PTPRC*), T cells (*CD3D*, *CD4*, *CD8A*), B cells (*CD19*), and MHC class II molecules (Supplementary Figure S3). The results suggested that the expression of DDX56 was associated with immune infiltration in the tumor microenvironment. Next, we estimated the proportions of immune cells in each TCGA tumor type by using CIBERSORT. We observed that *DDX56* expression was negatively correlated with immune infiltration levels of plasma cells, resting dendritic cells (DC), and CD4^+^ memory T cells (Figure 5B). Given that DDX56 participates in the immune infiltration process, it may have crucial biological functions in immunotherapy. Using transcriptome and clinical data from a recently published immunotherapy study (Liu et al., 2019b), we found that anti-PD-1-treated patients with high *DDX56* expression had shorter progression-free survival than those with low *DDX56* expression (Figure 5C).

### Pan-cancer analysis of genetic alteration, CNV, and methylation levels of DDX56

**FIGURE 6:**
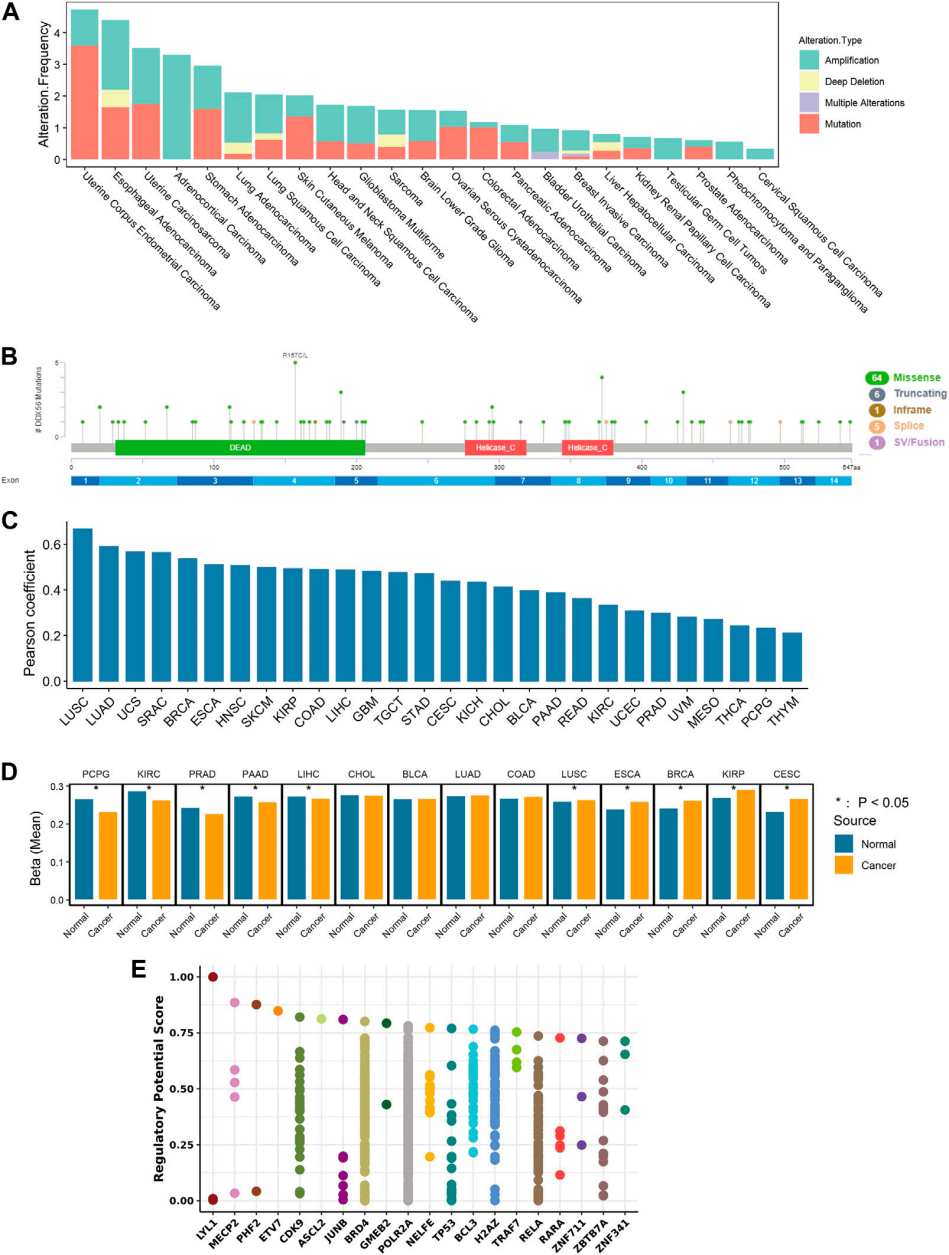
Mechanism of regulation of DDX56 expression. **(A)** Analysis of DDX56 alteration frequency in multiple cancer types, colored by mutation type. **(B)** Sites of different mutation types of *DDX56*. **(C)** Bar plot showing the Pearson correlation of CNV and *DDX56* RNA expression in different tumor types. **(D)** Bar plot showing the DNA methylation status of *DDX56* in tumors and normal tissues. *Y*-axis indicates the mean of the beta value. *p < 0.05. The statistics are from OncoDB. **(E)** Transcription factors potentially regulating *DDX56* (results obtained from Toolkit). The plot illustrates the top 20 factors. Dots on a single axis line indicate the same factor. Abbreviations: ACC: adrenocortical carcinoma; BRCA: breast invasive carcinoma; COAD: colon adenocarcinoma; GBM: glioblastoma multiforme; HNSC: head and neck squamous cell carcinoma; KICH: kidney chromophobe; KIRC: kidney renal clear cell carcinoma; LIHC: liver hepatocellular carcinoma; LUAD: lung adenocarcinoma; LUSC: lung squamous cell carcinoma; MESO: mesothelioma; OV: ovarian serous cystadenocarcinoma; PAAD: pancreatic adenocarcinoma; PCPG: pheochromocytoma and paraganglioma; PRAD: prostate adenocarcinoma; READ: rectum adenocarcinoma; SARC: sarcoma; SKCM: skin cutaneous melanoma; STAD: stomach adenocarcinoma; TGCT: testicular germ cell tumors; THCA: thyroid carcinoma; THYM: thymoma; UCEC: uterine corpus endometrial carcinoma; UCS: uterine carcinosarcoma; UVM: uveal melanoma.

The occurrence and development of cancer is related to gene alterations. To determine the genomic characteristics of DDX56 in cancers, comparative analysis of DDX56 was performed using cBioPortal. We observed that the main alterations of *DDX56* were amplifications and mutations in multiple tumors (Figure 6A). The most common mutation type was missense mutation, which could lead to alteration of protein structure and functions (Figure 6B). As CNV can lead to higher gene mRNA levels (Haraksingh and Snyder, 2013), we analyzed the correlation between RNA expression and CNV at the pan-cancer levels. The results showed that the CNV and RNA expression of DDX56 DNA in tumor tissues were significantly associated (Pearson coefficient >0.2, *p* < 0.05, Figure 6C). In addition, increased RNA transcription may result from low levels of DNA methylation (Bradner et al., 2017). We compared *DDX56* methylation status between various tumors and normal tissues and observed that *DDX56* methylation in five tumor types was lower compared with that in paired normal tissues (Wilcoxon test, *p* < 0.05) (Figure 6D). Furthermore, we estimated the correlation between expression and DNA methylation data using MEXPRESS. We observed that methylation of some CpG dinucleotides and CpG islands, including cg25257687 and cg01998345, was significantly negatively correlated with the RNA expression of *DDX56* (Pearson correlation coefficient <0 and *p* < 0.05, Supplementary Figure S4). Our results suggested that lower methylation levels of *DDX56* DNA may result in high expression levels of *DDX56* RNA in some cancers.

Finally, screening was performed to search candidate transcription factors possibly regulating *DDX56* expression. According to our results, *LYL1*, *MECP2*, *PHF2*, *ETV7*, and *CDK9* were the top five transcription factors with the potential to alter the expression level of DDX56 (Figure 6E). Furthermore, we estimated the co-expression relation of these top five transcription factors and *DDX56* (Spearman correlation, Supplementary Figure S5). We compared the expression levels of the top five transcription factors in tumor tissues and normal tissues (Wilcoxon test, Supplementary Figure S6). For example, we observed that the expression of CDK9 was highly related to the expression of DDX56 in THCA (Spearman correlation coefficient 0.384, *p* < 0.05, Supplementary Figure S5 and Supplementary Table S4). Simultaneously, we observed that the expression levels of CDK9 and DDX56 were both high in THCA tumor tissue (Wilcoxon test, *p* < 0.05, Supplementary Figure S6 and Figure 1A). We thus consider that CDK9 might be responsible for the high expression of DDX56 in THCA.

## Discussion

The role of DDX56 in tumorigenesis and development has attracted increasing attention in recent years (Zhu et al., 2020; Pryszlak et al., 2021; Sung et al., 2021; Wu et al., 2021). Although previous studies have reported upregulation of DDX56 in several tumor types, the underlying mechanisms of its pro-oncogenic function remain indistinct. Our results revealed that RNA expression of *DDX56* was indeed higher in 16 tumor types, which was confirmed at the protein level in nine tumor types. On this basis, we found that DDX56 may exert pro-oncogenic effects by enhancing proliferation and restraining apoptosis of tumor cells, affecting the infiltration of immune cells into the tumor microenvironment, and inducing tumor drug resistance. Our results suggest that DDX56 is involved in the occurrence and development of multiple cancers, as well as therapeutic response to chemotherapeutic agents. DDX56 could be an independent predictor of prognosis in a variety of tumor types and may also have value in prediction of immunotherapy efficacy.

Here, we report for the first time that the RNA and protein expression levels of DDX56 are significantly higher in tumor tissues than that in control normal tissues in various tumor types. Based on multi-omics data, we found that the high DDX56 expression in tumor tissues may be due to CNV and aberrant methylation. However, these changes were not present in all patients and may only partially explain the high DDX56 expression. Transcription factors are important components that regulate RNA transcription. We screened the potential transcription factors involved in regulating DDX56 using Toolkit. These transcription factors may be partially responsible for the abnormally high expression of DDX56, but more work needs to be done to reach such a conclusion.

We investigated the molecular mechanisms by which DDX56 promotes tumorigenesis and development through functional enrichment analysis. Our results suggested that DDX56 may promote the proliferation of tumor cells and inhibit tumor apoptosis. Zhu *et al.* showed that the knockdown of *DDX56* could reduce the proliferation and promoted the apoptosis of osteosarcoma cells (Zhu et al., 2020). Kouyama et al. (2019) showed that the overexpression of *DDX56* could enhance the proliferation of colon cancer cells. These results were consistent with our findings. Wu et al. (2021) further revealed that DDX56 could promote the proliferation of tumor cells through the WNT signaling pathway. Our results suggested that there may also be other molecular mechanisms involved, such as “E2F targets” and “MYC targets”, which have not previously been reported in DDX56-related studies. Therefore, we provide additional insights into the signaling pathways by which DDX56 promotes tumor proliferation.

Our functional enrichment results for DDX56 also suggested that DDX56 may be related to the infiltration of immune cells. We found that high *DDX56* expression was closely related to low infiltration levels of DC, plasma cells, and CD4^+^ T cells. DC are the most powerful antigen-presenting cells and can initiate immune responses (Gardner and Ruffell, 2016; Wculek et al., 2020). As critical mediators in anti-tumor immunity, plasma cells are capable of producing antibodies and CD4^+^ T cells secrete diverse cytokines that enhance humoral and cellular immunity (Borst et al., 2018; Sharonov et al., 2020). All of them are essential ancillary components in anti-tumor immunity, and their absence is detrimental to the immune reaction to cancer. This may also contribute to the poor prognosis and poor efficacy of immunotherapy in patients with high *DDX56* expression. Here, DDX56 was reported for the first time to be associated with infiltration of several immune cell types in the tumor microenvironment.

Combining all the data, we can conclude that DDX56 has a tumor-promoting function in most solid tumors. However, it may promote tumor progression in different ways in different cancer types. Therefore, we summarized the consistency of those aspects among different cancer types (Supplementary Table S3). For instance, there was no evidence that DDX56 could promote the proliferation of pheochromocytoma and paraganglioma (Figure 3A, *NSE*<0 or insignificant in all proliferation-related pathways). However, we observed that it was related to low level of apoptosis (Figure 5A, Supplementary Table S3) and could affect the infiltration of some immune cell types in pheochromocytoma and paraganglioma (Figure 5B, Supplementary Table S3).

Furthermore, we found that higher expression of DDX56 was associated with worse patient prognosis in multiple tumor types. High DDX56 expression was also found to related to lower efficacy of PD-1 antibody immunotherapy. All these results indicate that DDX56 could be used to predict not only prognosis but also the efficacy of immune checkpoint inhibitors. Our results also indicate that DDX56 may promote multiple anticancer drug resistance in tumor cells, which has not been previously reported.

We acknowledge several limitations of our study. Much more research needs to be done to determine whether DDX56 has the potential to predict prognosis and efficacy of chemotherapy drugs and immunotherapy. Although we has proposed potential pro-tumor molecular mechanisms involving DDX56 based on bioinformatics analysis, we did not perform biological experiments to validate these results. A large amount of CRISPR screens data verified our partial conjecture, giving some credibility to our results (Chow and Chen, 2018). However, the CRISPR screening evidence could not confirm the promoted proliferation ability of DDX56 and further functional experiments are needed to confirm the function of DDX56 and to explore the underlying mechanisms.

## Conclusion

In summary, we have clarified the tumor-promoting role of DDX56 based on multi-omics data at a pan-cancer level and elucidated the possible molecular mechanisms involved. These results contribute to our understanding of the biological function of DDX56 in tumors and provide evidence and potential research directions for future studies on DDX56 as an oncogenic driver.

## Data Availability

The datasets presented in this study can be found in online repositories. The names of the repository/repositories and accession number(s) can be found in the article/Supplementary Material.

## Supplementary material

Supplementary Figure S1 Comparison of DDX56 expression in populations with different clinical characteristics.

Supplementary Figure S2 Pan-cancer overall survival analysis of DDX56.

Supplementary Figure S3 Pan-cancer correlation analysis of DDX56 RNA expression with several cellular marker genes and MHC class II molecules.

Supplementary Figure S4 Correlation analysis of methylation levels of DDX56 DNA and expression levels of DDX56 RNA.

Supplementary Figure S5 Pan-cancer correlation analysis of DDX56 RNA expression with top five transcription factors screened by Toolkit (http://dbtoolkit.cistrome.org/).

Supplementary Figure S6 Bar plots showing median RNA expression levels of top five DDX56-related transcription factors in different tissues (Wilcoxon test). Significant: P<0.05; Insignificant: P>0.05 or P = 0.05.

Supplementary Table S1 Tumors involved in each analysis.

Supplementary Table S2 List of CRISPR screening studies showing that DDX56 is associated with the proliferation of cancer cells.

Supplementary Table S3 List of CRISPR screening studies showing that DDX56 is associated with drug resistance.

Supplementary Table S4 Results of pan-cancer correlation analysis of DDX56 RNA expression with top five transcription factors screened by Toolkit.

Supplementary Table S5 Summary of the results.
