# Supplementary figures and images for: Pan-cancer analysis identifies DDX56 as a prognostic biomarker associated with immune infiltration and drug sensitivity
Source: Front Genet. 2022 Dec 7;13:1004467. doi: 10.3389/fgene.2022.1004467 (PMC9768347; doi:10.3389/fgene.2022.1004467)

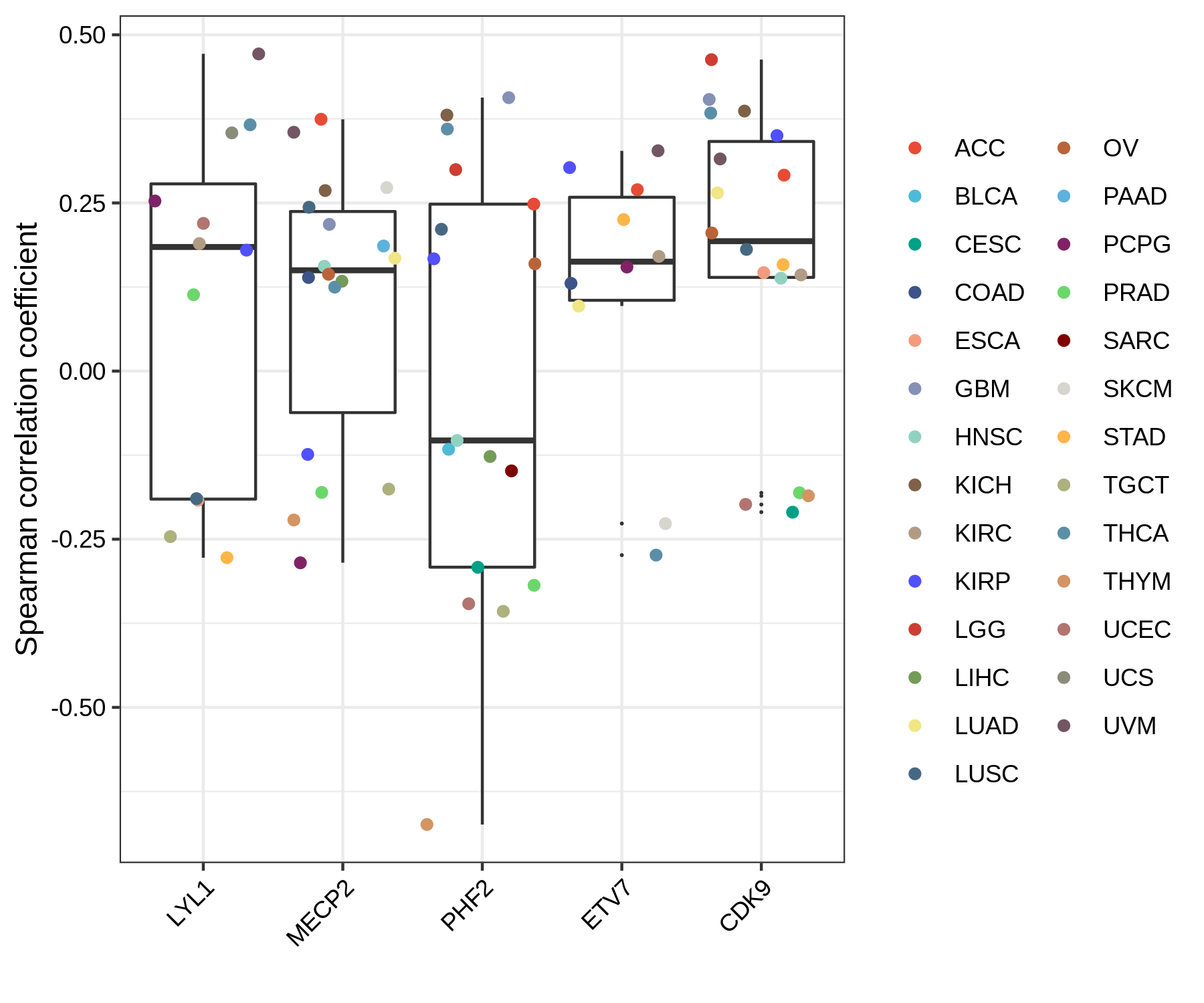

Supplement: Supplementary file 3 [file Image5.PNG]

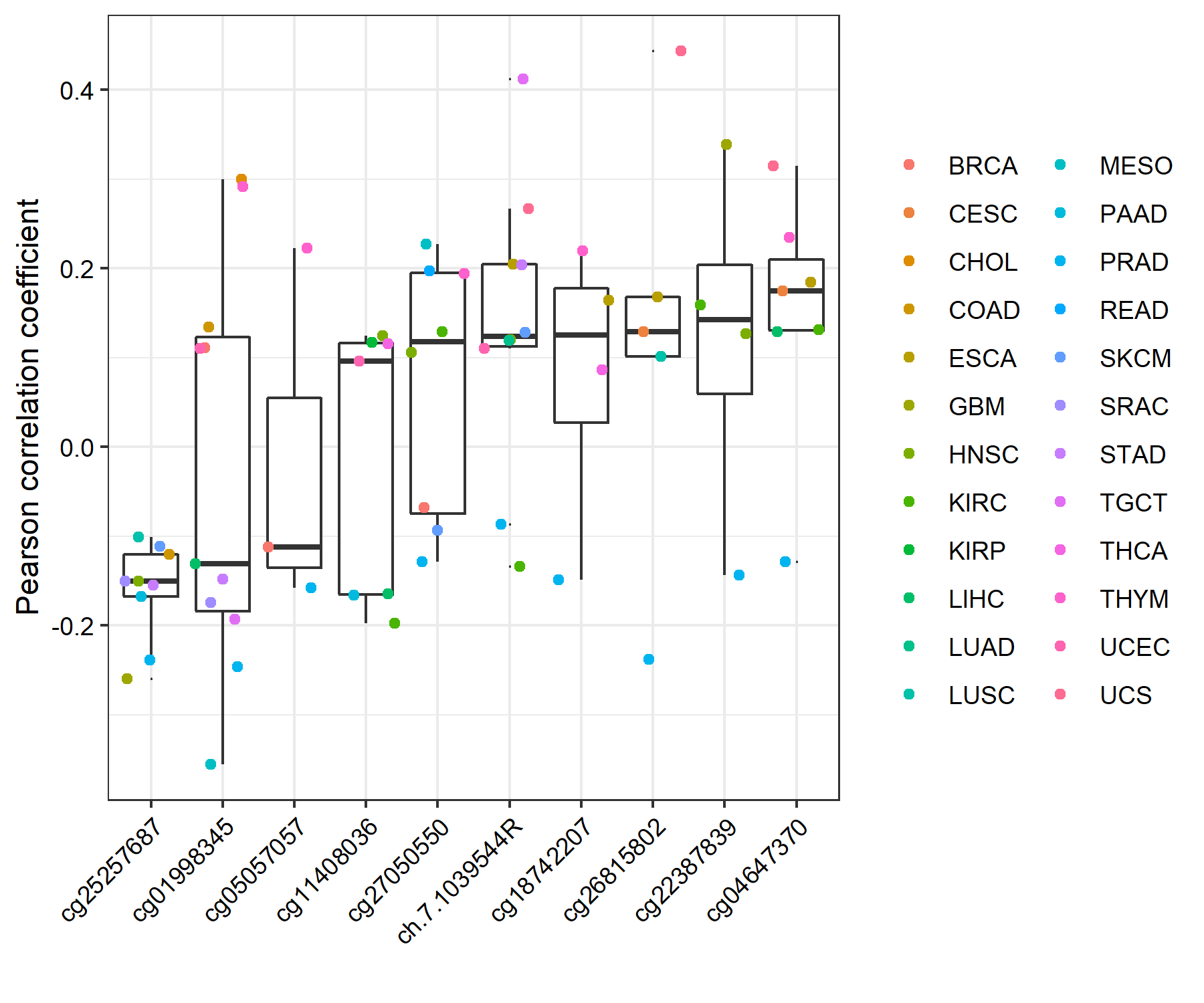

Supplement: Supplementary file 4 [file Image4.PNG]

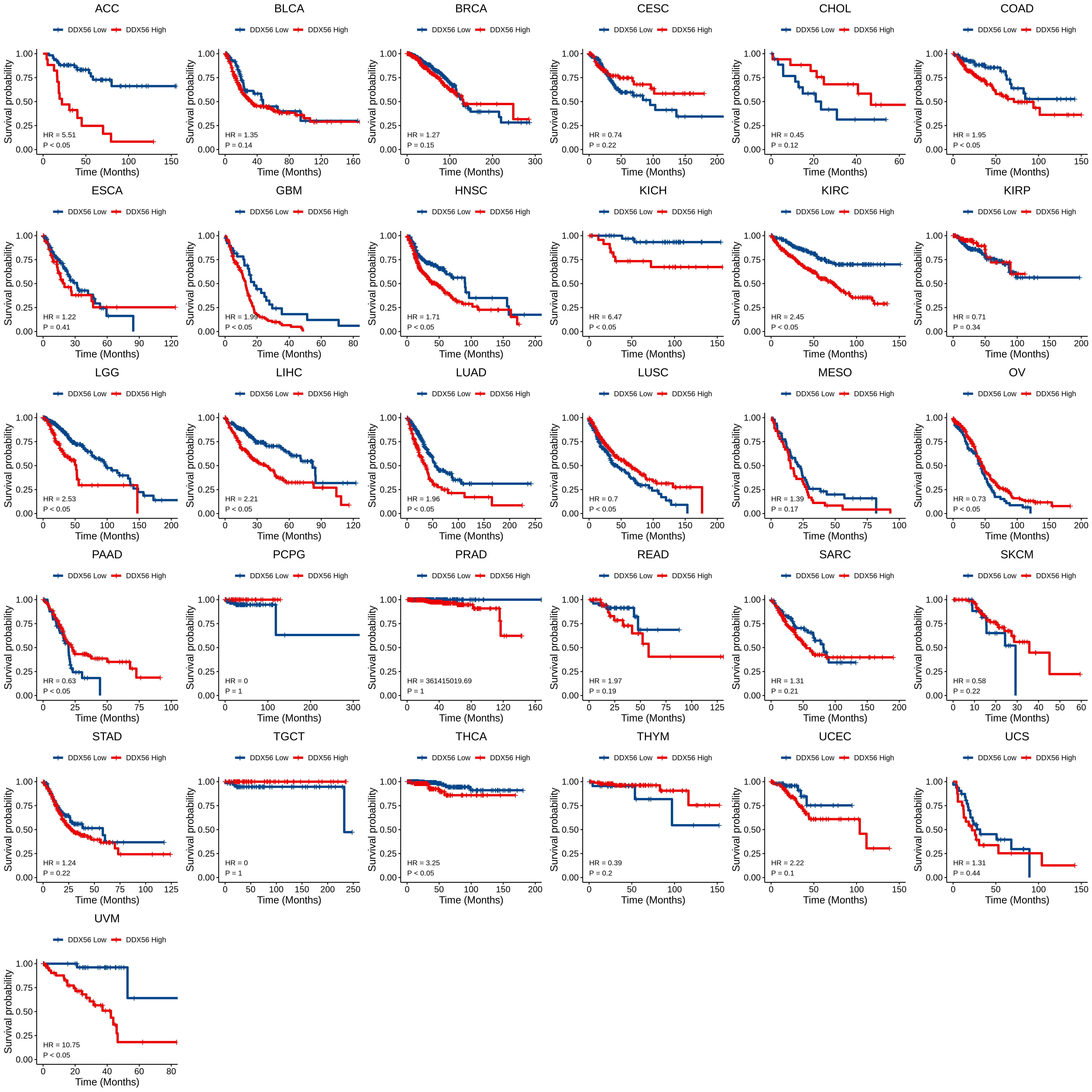

Supplement: Supplementary file 6 [file Image2.PNG]

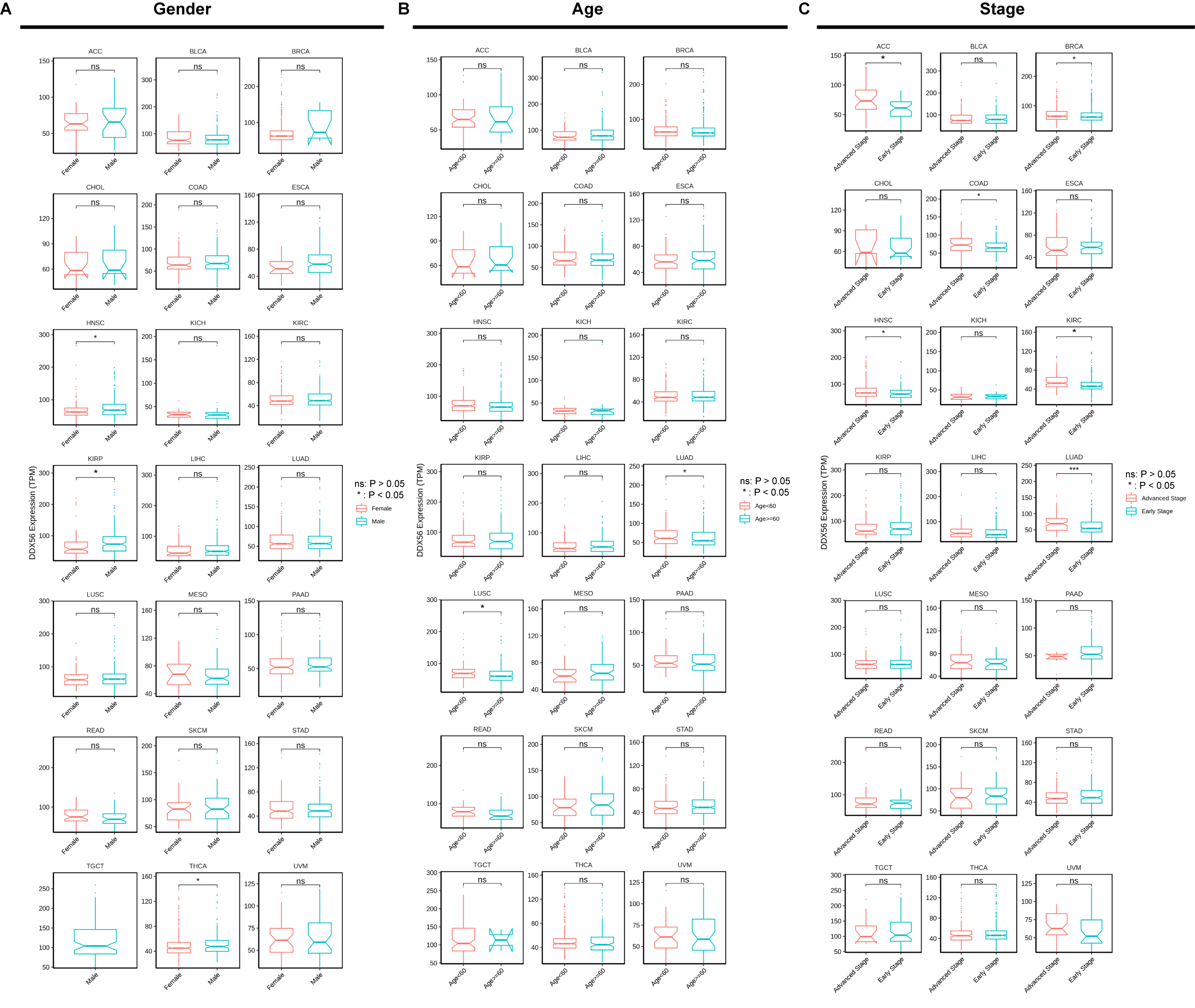

Supplement: Supplementary file 8 [file Image1.PNG]

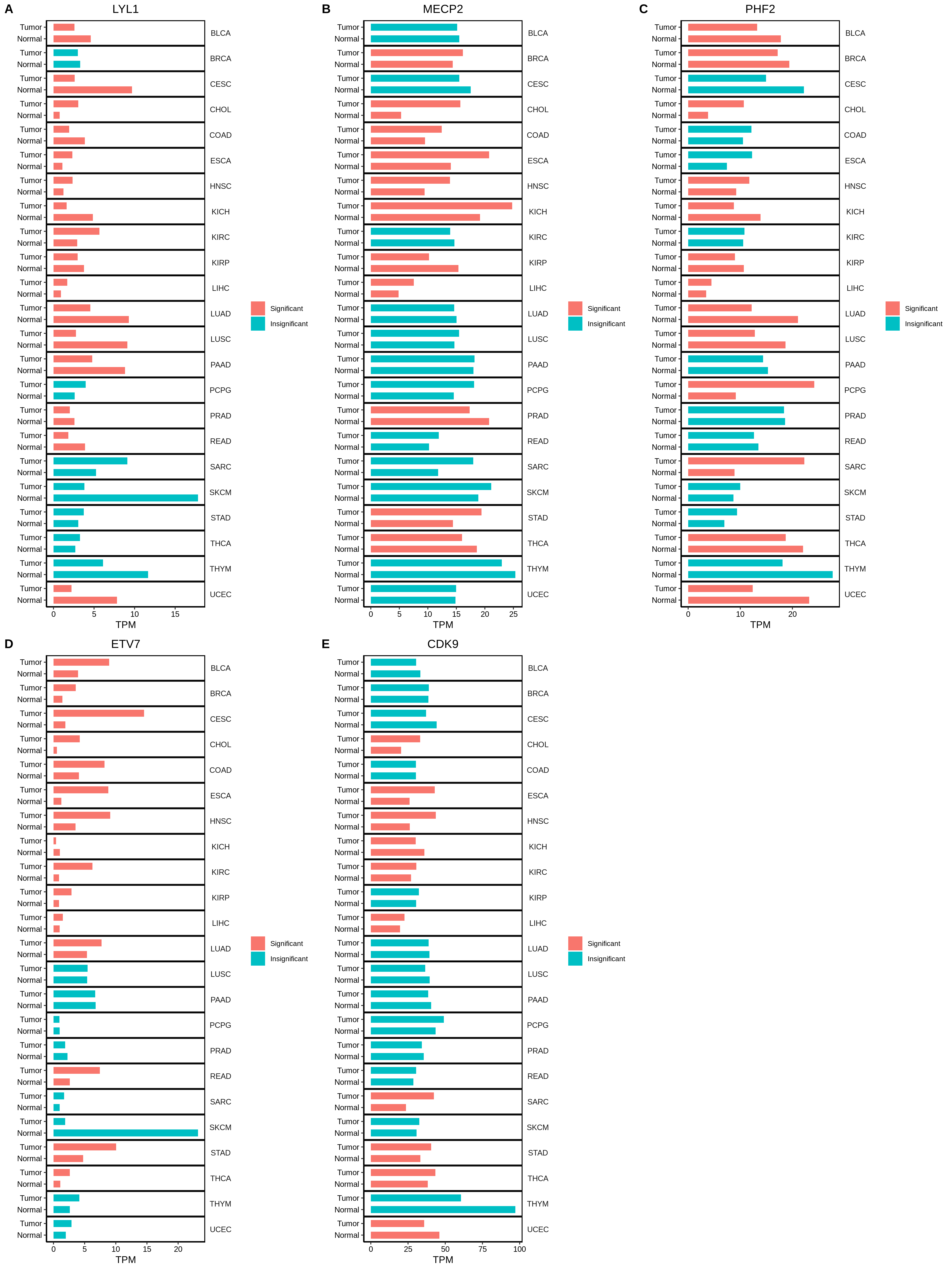

Supplement: Supplementary file 10 [file Image6.PNG]

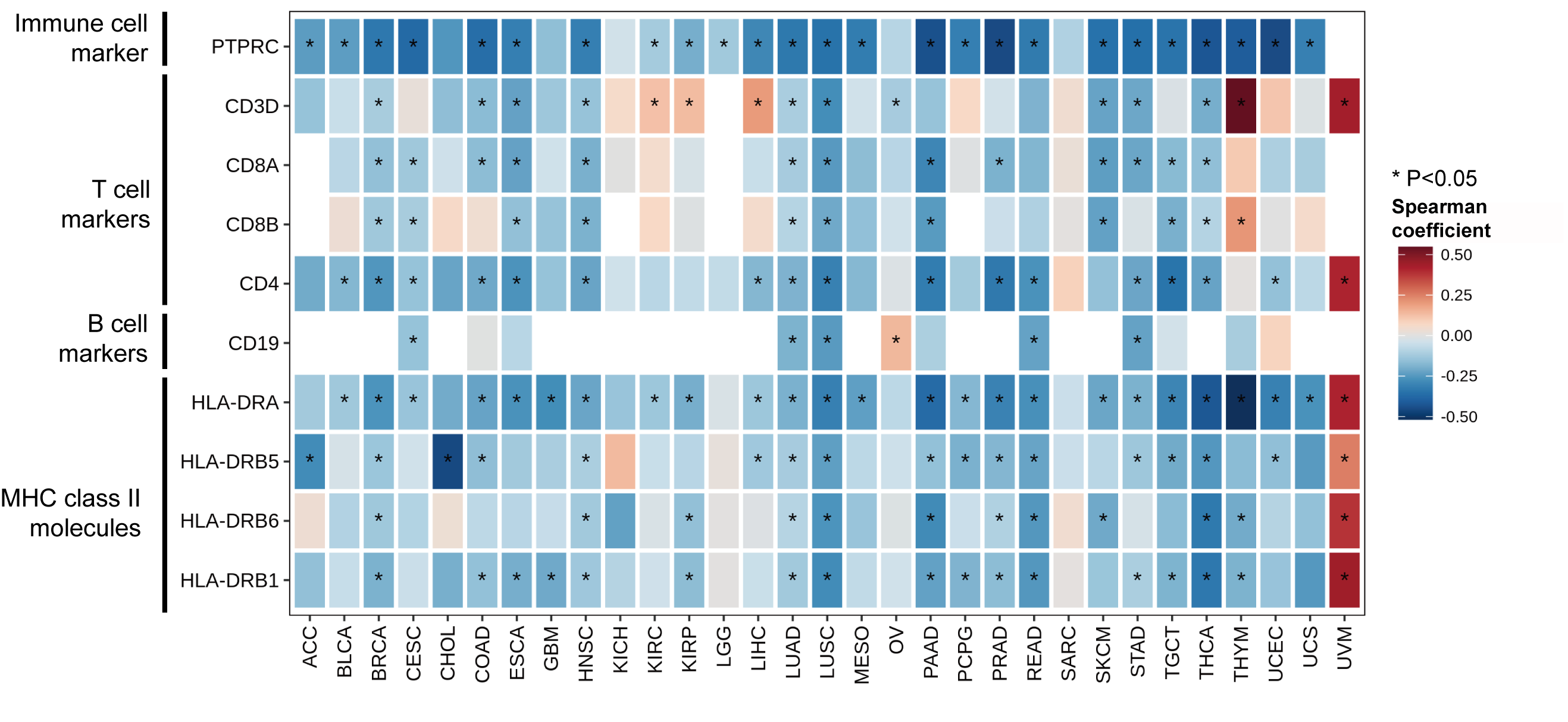

Supplement: Supplementary file 11 [file Image3.PNG]
